# Colorectal Cancer Is Borrowing Blueprints from Intestinal Ontogenesis

**DOI:** 10.3390/cancers15204928

**Published:** 2023-10-11

**Authors:** Jacob L. Billingsley, Veronika Yevdokimova, Kristina Ayoub, Yannick D. Benoit

**Affiliations:** 1 Department of Cellular and Molecular Medicine, University of Ottawa, Ottawa, ON K1H 8M5, Canada; jbill102@uottawa.ca (J.L.B.); vyevd006@uottawa.ca (V.Y.); kayou014@uottawa.ca (K.A.); 2 School of Pharmaceutical Sciences, University of Ottawa, Ottawa, ON K1H 8M5, Canada

**Keywords:** colorectal cancer, cancer stem cells, oncogenic reprogramming, Sox9, chemical genomics, differentiation therapy

## Abstract

**Simple Summary:**

A critical relationship was recently identified between fetal gene expression and aberrant stem cell properties in precancerous intestinal lesions. The reported findings show that developmental reprogramming, or the reactivation of genes expressed at the embryonic stage, precedes the initiation of colorectal cancer. Hence, harnessing such gene networks in future drug discovery endeavors has been proposed to suppress the initiation of colorectal tumors. In a recent drug discovery-oriented study, it was determined that stem-like functions in cancer, such as tumor initiation and self-renewal, can be suppressed by targeting gene signatures specific to germ layer commitment. Specifically, drugs with the capacity to induce endoderm, ectoderm, or mesoderm differentiation in human embryonic stem cells were also effective at suppressing the initiation of tumors from the same developmental origins. Altogether, the two cutting-edge studies showcased in this article reinforce the concept of oncogenic reprogramming as an early step in tumorigenesis. These studies are paving the way for new drug discovery efforts focused on suppressing fetal gene networks and fostering the differentiation of stem-like cells in colorectal cancer.

**Abstract:**

Colorectal tumors are heterogenous cellular systems harboring small populations of self-renewing and highly tumorigenic cancer stem cells (CSCs). Understanding the mechanisms fundamental to the emergence of CSCs and colorectal tumor initiation is crucial for developing effective therapeutic strategies. Two recent studies have highlighted the importance of developmental gene expression programs as potential therapeutic targets to suppress pro-oncogenic stem cell populations in the colonic epithelium. Specifically, a subset of aberrant stem cells was identified in preneoplastic intestinal lesions sharing significant transcriptional similarities with fetal gut development. In such aberrant stem cells, Sox9 was shown as a cornerstone for altered cell plasticity, the maintenance of premalignant stemness, and subsequent colorectal tumor initiation. Independently, chemical genomics was used to identify FDA-approved drugs capable of suppressing neoplastic self-renewal based on the ontogenetic root of a target tumor and transcriptional programs embedded in pluripotency. Here, we discuss the joint conclusions from these two approaches, underscoring the importance of developmental networks in CSCs as a novel paradigm for identifying therapeutics targeting colorectal cancer stemness.

## 1. Implications of Tumor Heterogeneity in the Initiation and Progression of Colorectal Cancer

Colorectal tumors are complex and heterogeneous cellular structures arising from a combination of genetic and epigenetic alterations [1,2]. The cellular heterogeneity within colorectal tumors contributes to therapeutic resistance, where most standard chemotherapy drugs tend to eliminate dividing cell populations and select for resistant subsets [1]. As with most human neoplasms, the cellular heterogeneity of colorectal tumors contains a small fraction of self-renewing entities capable of tumor initiation that are termed cancer stem cells (CSCs) [3]. Colorectal CSCs represent a substantial challenge for clinical oncology as these are notoriously famous for evading standard debulking chemotherapeutics through intrinsic or adaptive resistance mechanisms and by promoting an immune cold tumor microenvironment [1,4].

The emergence of CSCs in the early steps of carcinogenesis relies on the reprogramming of the epigenome, which is marked by changes in the activation state of key chromatin regulators re-wiring gene expression networks [5,6,7]. For instance, genes encoding epigenetic modifying enzymes, including DNA (cytosine-5)-methyltransferase 3A (DNMT3A) and isocitrate dehydrogenase IDH1/2, were mutated at a high frequency in the founding clones of human acute myeloid leukemia (AML) [8,9]. Inactivating mutations in DNMT3A were identified as precursors of leukemogenesis and the characteristics of pre-leukemic stem cells in a subset of patients [10]. In colorectal cancer, members of the Polycomb group machinery B cell-specific Moloney murine leukemia virus integration site 1 (Bmi1) and enhancers of zeste homolog 2 (EZH2), as well as histone methyltransferase G9a, are overexpressed and critical for the maintenance of CSC functional hallmarks [11,12,13]. Specifically, G9a and EZH2 were both implicated in the maintenance of pluripotent-like gene expression signature in colorectal tumor-initiating cells [11,13]. Given the bendable nature of chromatin organization, it is evident that CSCs represent a cell state rather than a definite cell type and that cell plasticity plays a central role in maintaining an equilibrium between stem and non-stem units across heterogeneous malignant tissues [7,14].

In colorectal cancer, the relationship between CSCs and cell plasticity was highlighted in lineage-tracing experiments using murine models where selective ablation of CSCs based on the intestinal stem cell marker leucine-rich repeat-containing G-protein coupled receptor 5 (Lgr5) was performed [15,16]. Upon the ablation of Lgr5^+^ cells, tumors were maintained by the proliferative capacity of Lgr5-negative cells. These can eventually restore the pools of Lgr5^+^ CSCs, together with sustained tumor growth and liver metastasis [15]. Interestingly, most colorectal cancer circulating metastasis-initiating cells are LGR5-negative despite holding clear tumor-initiating capacities [16]. LGR5^+^ cells eventually appear via cell plasticity in nascent metastases and are indispensable in maintaining tumor growth. Such an implication of LGR5-negative cells in colorectal cancer dissemination points to the limitation of using protein markers to assess CSC activity. The clonogenic serial in vivo repopulation assay remains the gold standard approach to measure CSC functions like self-renewal and tumor initiation [17].

## 2. An Aberrant Stem Cell (AbSC) Population Characterized by a Fetal-like Gene Signature Drives the Initiation of Intestinal Neoplasia

**Figure 1:**
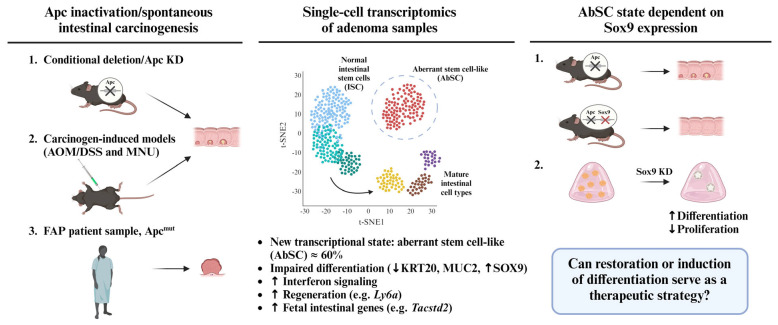
Identification of aberrant stem cell-like population driving colorectal cancer initiation. A subset of aberrant stem-like cells (AbSCs) was identified from mouse and human intestinal adenoma tissues using single-cell transcriptomics. (Lgr5^Cre^;Apc^f/f^;R26^tdT^ (Apc^KO^) and Apc knockdown (KD) mice. AOM/DSS and N-Methyl-N-nitrosourea (MNU) carcinogenic treatments. Primary adenoma tissues from a familial adenomatous polyposis (FAP) patient.) The AbSC population was characterized by the impaired (↓) differentiation marker expression (KRT20, MUC2) and reactivation (↑) of fetal intestinal genes, as well as transcriptional programs of regeneration and aberrant interferon signaling. The AbSC cluster shows an increased level (↑) of Sox9, which is required for the initiation of intestinal neoplasia. Sox9 silencing in Apc^KO^ model prevented the formation of adenomatous polyps, which was further confirmed by Sox9 knockdown in FAP-derived organoids, where increased (↑) differentiation and reduced (↓) proliferation levels were observed. The findings herein summarized highlight the potential for differentiation therapy targeting fetal-like molecular signatures in colorectal cancer.

In a recent publication, Bala et al. used single-cell transcriptomics (scRNA-seq) on digestive epithelium tissues from genetic deletion and chemically induced models of colorectal tumorigenesis to depict critical molecular events, leading to premalignant intestinal lesions [18]. This study identified SRY-box 9 (Sox9) activation as a common feature of tumor initiation in all the tested models. While Sox9 is normally confined at the bottom of the intestinal crypt compartment where intestinal stem cells reside, this HMG-box transcription factor is strongly expressed in poorly differentiated lesions and early-stage adenomas. scRNA-seq performed on intestinal epithelium cells conditionally deleted for Apc (vs. wild type) revealed the existence of a subpopulation of aberrant stem cells (AbSCs) sharing transcriptional similarities with healthy intestinal stem cells, but marked by a unique fetal-like intestinal gene expression signature (Figure 1).

Notable among the genes upregulated in the AbSC signature is tumor-associated calcium signal transducer 2 (Tacstd2), which encodes the transmembrane glycoprotein Trop2 and is associated with stem-like progenitor phenotypes (Figure 1) [19]. Tacstd2 was found to be overexpressed in different types of epithelial tumors and identified as an important driver of neoplastic self-renewal in prostate cancer, following the cleavage of its intracellular domain [19]. In organoid models of intestinal-like gastric dysplasia, Tacstd2 knockdown reduced the growth and formation of budding structures typically associated with stem cell-containing crypt-like organization [20,21].

The gene expression profile characterizing the AbSC subset was also observed in colonic lesions from azoxymethane and dextran sulfate sodium (AOM/DSS)-treated mice, but not in healthy adjacent mucosa (Figure 1) [18]. Ultimately, the AbSC and fetal-like intestinal gene signatures were found to be enriched in a cluster of precancerous adenoma cells presenting abnormal gene expression (vs. paired healthy sample) isolated from a patient with familial adenomatous polyposis (FAP) syndrome (Figure 1). In all cases, the AbSC fractions showed impaired differentiation, as denoted by a reduction in the expression of differentiation markers keratin 20 (Krt20) and mucin 2 (Muc2), paired with elevated Sox9 expression (Figure 1). Importantly, the transcriptional state of the AbSC population, uniquely characterized by the upregulation of genes associated with interferon signaling, regeneration, as well as expression of fetal intestinal genes, was distinct from normal intestinal stem cells; it was therefore referred to by the authors as an aberrant rather than a hyperactive stem-like state (Figure 1).

## 3. Sox9 Expression Regulates Developmental Reprogramming and Maintenance of AbSCs

One of the key findings made by Bala et al. highlights the role of Sox9 as a regulator of the AbSC population. Specifically, the ablation of Sox9 in adenomatous polyposis coli (Apc)-deleted mice reduced the AbSC frequency, and, therefore, in vivo adenoma formation (Figure 1). Further, the scRNA-seq of the intestinal epithelia from these mice revealed that multilineage differentiation capacities were restored upon Sox9 knockdown. While Apc knockout (Apc^KO^) resulted in 60% of stem cells expressing the AbSC signature, the decreasing Sox9 expression reduced this population by six-fold. Consistent with findings in mice, multilineage differentiation capacities were restored upon Sox9 knockdown in adenoma organoids derived from APC-mutant human colonic tissues (Figure 1). 

Integrative multi-omic analysis confirmed the pivotal role of Sox9 in the reprogramming of chromatin accessibility and the induction of developmental gene expression programs in the intestinal epithelium of Apc^KO^ mice. Specifically, the authors used an assay for transposase accessible-chromatin sequencing (ATAC-seq) to demonstrate that Apc^KO^ cells within the murine intestinal epithelium exhibit higher chromatin accessibility in genes associated with regenerative and fetal processes, such as lymphocyte antigen 6 family members A and E (Ly6a, Ly6e). By conditionally deleting Sox9 in mouse Apc^KO^ intestine, Bala et al. observed a significant reduction in chromatin accessibility for the genes involved in regenerative functions and fetal-like programming, including Ly6a, Ly6e, and tumor-associated calcium signal transducer 2 (Tacstd2) [18].

Sox9 was previously reported as a driver of CSC functions in colorectal cancer through a genome-wide enhancer enrichment pattern promoting stem-like transcriptional programs [22]. In this study, higher Sox9 protein levels were observed in human intestinal dysplasic lesions and adenocarcinoma compared to those of normal adjacent tissue. Silencing the Sox9 expression in different colorectal cancer models resulted in a loss of clonogenic functions, together with reduced proliferation and the increased expression of intestinal differentiation markers. Using mouse Apc^KO^ intestinal organoids, it was shown that Sox9 inhibition results in a significant downregulation of the colorectal CSC marker CD133/Prominin-1. Additionally, knocking down Prominin-1 in LS180 cells reduced the Sox9 expression level, suggesting the existence of an Sox9-Prominin-1 feedback loop in colorectal CSC-like cells. Overall, the genomic experiments presented by Bala et al., together with functional experiments from Liang et al., robustly support the role of Sox9 in colorectal CSC-related functions [22].

The relationship between CSCs and developmental-like reprogramming was previously documented in colorectal cancer, where epigenetic regulators such as histone methyltransferases EZH2 and G9a, which are vital to embryonic patterning, were tied to self-renewal and tumor initiation capacities [11,13]. The pharmacological inhibition of such chromatin-modifying enzymes was proposed as a new therapeutic avenue to block colorectal CSC functions. Hence, the novelty of the findings highlighted by Bala et al. lies in how development-like programs are integrated in intestinal precancerous lesions. Considering their essentiality to the onset of aberrant stem-like gene expression and subsequent tumor initiation in the colonic epithelium, development-associated factors (e.g., Sox9) are now also proposed as prospective therapeutic targets to block tumorigenesis at the root (Figure 1).

Akin to Bala et al., a study by Kopp and colleagues suggested, more than a decade earlier, that targeting Sox9 expression might represent a powerful approach to restrict cell plasticity in premalignant lesions, leading to pancreatic ductal adenocarcinomas [23]. In this context, Sox9 overexpression contributed to acinar-to-ductal reprogramming following pancreatic injuries (e.g., pancreatitis) and was found to be essential to progression toward pancreatic intraepithelial neoplastic lesions (PanIN). Interestingly, in the immunohistochemical analysis of Sox9 expression using tissue microarrays, both chronic pancreatitis tissue and low-grade PanINs were found to be generally Sox9-positive, while in higher-grade pancreatic lesions, the Sox9 expression was more heterogeneous. Such observations suggested that Sox9 expression is associated with the initiation of pancreatic ductal tumorigenesis. In a mouse model of pancreatic carcinogenesis, the concomitant induction of the Kras^G12D^ oncogenic mutant expression and the deletion of Sox9 in acinar cells, which, unlike ductal cell, do not normally express Sox9, prevented the formation of PanINs and maintained a normal pancreatic morphology. Consistent with these findings, the overexpression of Sox9 in acinar cells in the context of Kras^G12D^ mutation rapidly induced the formation of pancreatic lesions. Thus, it was shown that Sox9 activity in acinar cells was critical to their transition toward a duct-like phenotype via cellular plasticity and required for the induction of PanINs.

## 4. Chemical Induction of Germ Layer Commitment Programs in Aberrant Stem Cells Is a Promising Approach to Treat Colorectal Cancer and Other Malignancies

The findings by Bala et al. point to the existential question of whether differentiation therapy aiming at exhausting neoplastic stem cells is a viable therapeutic avenue to treat colorectal cancer [18]. Orlando et al. recently identified a fascinating relationship between tumors from the same ontogenetic root and their responsiveness to chemical inducers of early embryonic differentiation programs [24]. Single-cell transcriptomics performed by Orlando et al. enabled the deconstruction of human pluripotent stem cell (hPSCs) heterogeneity, which is marked by uncommitted and germ layer-primed clusters. By comparing genes significantly upregulated in the mouse Apc^KO^ AbSC cluster across hPSCs heterogeneity, we observe a four-fold higher overlap between AbSC and “uncommitted” hPSC genes than that of the “primed” clusters [18,24].

Thus, Orlando et al. used an hPSC-based high-throughput chemical genomic pipeline to identify FDA-approved drugs with the capacity to suppress neoplastic self-renewal. This first step of their screening campaign was performed on a transformed variant of hPSCs previously documented as a surrogate model of cancer stem cells, exhibiting a differentiation blockade and enhanced self-renewal properties [11,25,26]. The pluripotency factor OCT4 was used as a readout through this initial screening step since the repression of such key transcription factor represents one of the earliest events in the progress away from stemness [27]. A similar rationale was used to identify and characterize other CSC-impacting drugs, such as thioridazine, as well as the reverse-turn peptidomimetic compounds CWP232228 and YB-0158 [25,27,28].

The compounds inhibiting neoplastic self-renewal were then tested for their ability to induce germ layer-specific differentiation programs in hPSCs. Specific chemical inducers of endoderm, ectoderm, and mesoderm fates were identified [24]. Astonishingly, the drugs inducing endoderm differentiation in hPSCs were the best at stimulating differentiation marker expression in human colorectal cells, while mesoderm and ectoderm inducers efficiently restored differentiation in leukemia and glioblastoma cells, respectively. When it was tested using patient-derived tumor organoid formation assays, the BET bromodomain inhibitor hexamethylene bisacetamide (HMBA) [29], which robustly induced endoderm commitment in hPSCs, selectively suppressed tumor initiation in colorectal tumor tissues. In contrast, HMBA did not affect human leukemic progenitor activity and primary glioblastoma tumoroid formation [24]. Further transcriptomic analyses revealed that HMBA selectively alters the gene expression programs commonly shared between hPSCs and other tumors from endoderm ancestry, including prostate and esophageal cancers.

Similar observations were made for the compounds that induced mesoderm and ectoderm fate commitment in hPSCs when applied to cancer models, with respect to the ontogenetic roots. The ectoderm-inducing compounds identified by Orlando and colleagues triggered astrocyte differentiation marker expression in the human glioblastoma multiforme (GBM) cell line U87MG, while endo- and mesoderm-inducers displayed no significant effects [24]. One of the three drugs inducing ectoderm differentiation, the breakpoint cluster region-ABL1 fusion (BCR-ABL) inhibitor AP-24534, substantially reduced stem/progenitor activity in patient-derived GBM spheroid assays. In leukemia, only treatments with mesoderm inducers increased the expression of the differentiation marker CD11b in the NB4 cell line, and two out of three identified mesoderm inducers inhibited leukemic progenitor function in patient-derived AML samples. The two compounds effectively targeting leukemic progenitor functions were GW843682X, an inhibitor of polo-like kinase 1/3 (PLK1/3), and ABT-263, also known as navitoclax, a B-cell lymphoma 2 (BCL-2) inhibitor. Interestingly, another BCL-2 inhibitor, venetoclax, has been approved for the treatment of AML and other leukemias, providing clinical validation of the proposed differentiation-based treatment strategy [30,31]. Importantly, mesoderm inducers identified by Orlando et al. had no impact on the clonogenic activity of hematopoietic progenitors from healthy donors. Altogether, these findings highlight a unique overlap between molecular programs rooted in early development and adult malignancies, but not in normal tissue homeostasis [24].

Building on the above-described concepts, Orlando et al. characterized the gene networks commonly involved in germ layer specification in hPSCs and the induction of differentiation programs in human cancers. Transcriptome profiling after being given treatments with germ-layer-inducing compounds revealed a previously overlooked role of metallothionein family 1 (MT1) genes in mesodermal fate specification. Additionally, MT1 genes were found to be overexpressed in AML and other cancers originating from mesoderm, such as ovarian and renal tumors. Similarly, the dual specificity phosphatase 6 (DUSP6) was identified as a barrier gene to endoderm commitment in hPSCs and found to be overexpressed in human GBM. In both cases, the knockdown of MT1s and DUSP6, respectively, enhanced the differentiation marker expression level in the human leukemia and GBM models, supporting their relevance as potential targets in cancer differentiation therapies.

## 5. Discussion

Altogether, the findings from Orlando et al. point toward a novel paradigm for identifying therapeutics targeting stemness in cancer, which lies within early developmental networks, as suggested by Bala et al. The concept of the reactivation of embryonic gene expression in cancer has evolved for more than two decades, from studies focused on singular genes and signaling pathways along with histological observations [32,33] to systematic analyses of transcriptional networks, providing a deeper understanding of the mechanisms of oncogenic reprogramming, eventually coming to an age of potential therapeutic translation. In a pioneering transcriptomic study, Ben-Porath et al. linked the preferential expression of genes enriched in embryonic stem cells (ESCs) with histologically poorly differentiated, high-grade aggressive tumors [34]. The high-level expression of gene sets associated with ESC identity, such as Nanog, Oct4, Sox2, and Myc targets, and the underrepresentation of the expression of Polycomb target genes were observed in poorly differentiated tumors of various origins (breast cancer, glioblastoma, and bladder carcinoma). Importantly, it was established that the phenotype of tumor-initiating cells is rooted in the hPSC-like transcriptional state, rather than the general state of rapid proliferation. Moreover, Kim et al. argued that the main signature accounting for similarities between ESCs and cancer cells is a Myc-centered regulatory network, rather than a core pluripotency transcription program [35].

Later, Lin and colleagues summarized the targetable developmental mechanisms that play a role in cancer progression, which include not only self-renewal and differentiation arrest, but also immune evasion and the reactivation of primordial mechanisms of motility enabling invasion and metastasis, all of which are also influenced by microenvironmental cues [36]. With the capacity of machine learning to integrate complex transcriptomic data, Malta et al. developed an algorithm for assessing the degree of oncogenic dedifferentiation, which they termed Stem Cell Index calculation [6]. DNA methylation- and mRNA expression-based stemness indices were shown to be correlated with tumor pathology and capable of predicting patients’ clinical outcomes. Importantly, using tumor samples with the highest and lowest Stem Cell Index levels and Connectivity map (CMap) analysis, Malta and colleagues were able to identify drugs that were shown to inhibit cancer stemness [6].

As an example of a promising targetable developmental pathway in the context of colorectal CSCs, the chemical inhibition of the histone methyltransferase G9a downregulated the expression of ESC-associated genes and Myc target signatures in human CSC models [11]. Such observations were supported by a decrease in the Stem Cell Index following treatments with G9a inhibitors. Ultimately, the alteration of pluripotent-like gene signatures via blocking G9a activity reduced tumor-initiating activity and restored intestinal differentiation in human colorectal cancer models, supporting the concept of targeting embryonic roots of tumors as a promising therapeutic strategy.

## 6. Conclusions

The elegant in vivo demonstration of developmentally rooted, aberrant stem cell activity preceding colorectal tumor initiation by Bala et al., together with the extensive chemical genomics and patient-derived functional assays deployed by Orlando et al. represent a solid argument for new therapeutics designed against embryonic networks in CSCs. Merging the conclusions from both studies represents an important step in addressing the current limitations of conventional drug discovery endeavors aiming to target tumor heterogeneity and self-renewal by giving further importance to the developmental origin of each cancer.

## Data Availability

No original data were reported.
